# 

**Published:** 1911-06

**Authors:** 


					CONTENTS. I UWPS, A
On the Resection of the Posterior Spinal Nerve Roots. 1
ji'ETy
Ernest W. Hey Groves, M.S.Lond., F.R.C.S. ...
,r>'
Some Observations on School Hygiene.
By J. O. Symes, M.DTLond..
D.F.H.
log
Design for an Elementary Radiographic Camera.
By William
Cotton, M.D. Edin.
n8
The Medical Aspect of Boswell's "Life of Johnson, with some
Account ot the Medical Men mentioned in that Book.
Bertram M. H. Rogers, M.D., B.Ch., B.A. Oxon.
125
Auricular Fibrillation.
By C. E. K. Herapath, M.D.Lond. ...
148
Furunculosis.
By L. Shingleton Smith, M.B.Cantab....
157
Four Cases of Cysts in the Neck of Similar Character, and
Possibly Developed in connection with Branchial Clefts.
By
Charles A. Morton, F.R.C.b.
i6o
A Case of Ataxia in a Child aged three and a halt years.
By ilDWARD
Cecil Williams, B.A., M.B.Cantab.
i6i
Progress of the Medical Sciences :
Medicine.
T. M. Fortescue-Brickdale, M.A., M.13., B.Ln.Oxon.
i65
Surgery.
H. G. Kyle, M.B., B.Ch. Oxon.
i6g
Reviews of Books
i7i
Editorial Notes
IOO
Notes on Preparations for the Sick
185
The Library of the Bristol Medico-Chirurgical Society   188
Meetings of Societies
igi
Obituary Notices
195
Local Medical Notes
igg

				

